# *Lentilactobacillus kefiri* SGL 13 and *Andrographis paniculata* alleviate dextran sulfate sodium induced colitis in mice

**DOI:** 10.3389/fnut.2023.1072334

**Published:** 2023-02-13

**Authors:** Laura Manna, Eleonora Rizzi, Eleonora Bafile, Andrea Cappelleri, Massimiliano Ruscica, Chiara Macchi, Michele Podaliri Vulpiani, Romolo Salini, Emanuela Rossi, Concetta Panebianco, Francesco Perri, Valerio Pazienza, Federica Federici

**Affiliations:** ^1^PNK Farmaceutici S.p.a., Castelnuovo Vomano, Italy; ^2^Sintal Dietetics S.r.l., Castelnuovo Vomano, Italy; ^3^Department of Veterinary Medicine (DIMEVET), University of Milan, Milan, Italy; ^4^Mouse and Animal Pathology Laboratory (MAPLab), Fondazione UNIMI, Milan, Italy; ^5^Department of Pharmacological and Biomolecular Sciences, University of Milan, Milan, Italy; ^6^Istituto Zooprofilattico Sperimentale dell’Abruzzo e del Molise “Giuseppe Caporale” (IZSAM), Teramo, Italy; ^7^Division of Gastroenterology, Fondazione IRCCS-Casa Sollievo della Sofferenza, Foggia, Italy

**Keywords:** *Lentilactobacillus kefiri*, *Andrographis paniculata*, inflammatory bowel disease, dextran sodium sulfate, colitis, inflammatory mediators, dysbiosis, gut microbiota

## Abstract

**Introduction:**

Inflammatory bowel diseases (IBD) are chronic inflammatory conditions that typically involve diarrhea, abdominal pain, fatigue, and weight loss, with a dramatic impact on patients’ quality of life. Standard medications are often associated with adverse side effects. Thus, alternative treatments such as probiotics are of great interest. The purpose of the present study was to evaluate the effects of oral administration of *Lentilactobacillus kefiri* (basonym: *Lactobacillus kefiri*) SGL 13 and *Andrographis paniculata*, namely, *Paniculin 13*™, on dextran sodium sulfate (DSS)- treated C57BL/6J mice.

**Methods:**

Colitis was induced by administering 1.5% DSS in drinking water for 9 days. Forty male mice were divided into four groups, receiving PBS (control), 1.5% DSS, *Paniculin 13*™ and 1.5% DSS + *Paniculin 13*™.

**Results:**

The results showed that body weight loss and Disease Activity Index (DAI) score were improved by *Paniculin 13*™. Moreover, *Paniculin 13*™ ameliorated DSS-induced dysbiosis, by modulating the gut microbiota composition. The gene expression of MPO, TNFα and iNOS in colon tissue was reduced and these data matched with the histological results, supporting the efficacy of *Paniculin 13*™ in reducing the inflammatory response. No adverse effects were associated to *Paniculin 13*™ administration.

**Discussion:**

In conclusion, *Paniculin 13*™ could be an effective add-on approach to conventional therapies for IBD.

## Introduction

Inflammatory bowel disease (IBD) is a chronic inflammatory disorder of the gastrointestinal tract that refers mainly to Crohn’s disease (CD) and to ulcerative colitis (UC) (1). The intestinal inflammation is the result of a combination of immune system disorders, genetic susceptibility, environmental factors, and imbalance of the normal gut microbiota (2, 3). The etiology of inflammatory bowel disease is still unknown, but the main assumption is that the inflammation is triggered by the altered and pathogenic microbiota in genetically prone subjects. In fact, mouse model studies of IBD demonstrated protection against the development of the pathogenesis in germ free intestinal tract animals, supporting the role of gut flora in the onset of symptoms (4). Therapeutic approaches for IBD include anti-inflammatory, immunosuppressive, and biologic therapies, largely prescribed in clinical practice (5). Due to its chronic nature, management of IBD generally requires long-term treatment which often results in side effects or discomfort. Moreover, many patients with IBD are refractory to conventional therapies (6) requiring for alternative treatments. In this context, the role of microbiota can not be underestimated (7). Indeed, a significant difference in the gut microbiome of healthy subjects and IBD patients has been described (8, 9). The pattern of dysbiosis most often associated with IBD is a decrease in commensal bacteria diversity, mostly *Firmicutes* and *Bacteroides*, and a relative raise of bacterial species belonging to *Enterobacteriaceae* (10, 11). Therefore, the modulation of intestinal microbiota, such as by means of fecal bacteria transplantation (FMT) or pro/prebiotics, can restore a healthy gut composition. This represents a valuable alternative and an effective therapeutic option to treat IBD (12).

Among IBD patients, probiotics have gained a great interest due to their feature to alleviate clinical symptoms and to improve the quality of life, either during periods of exacerbation or remission (13–15). In animal model, different strains of lactic acid bacteria, e.g., *Bifidobacterium longum* (16), *Lactococcus lactis* (17), *Lactobacillus plantarum* (recently reclassified as *Lactiplantibacillus plantarum*) (18), *Lactobacillus reuteri* (recently renamed *Limosilactobacillus reuteri*) (19) have been reported to modulate the gut microbiota composition leading to a restored intestinal barrier function.

In clinical trials enrolling individuals with IBD, several probiotic strains have alleviated intestinal mucosal inflammation, have reduced colonic myeloperoxidase and fecal calprotectin levels, and have delayed the in-between symptom recurrences (20, 21).

Moreover, natural products derived from plants and herbals, are also increasingly used by IBD patients (22). *A. paniculata*, a traditional Chinese medicine with anti-inflammatory, antimicrobial and immunomodulatory properties, is often used to treat gastrointestinal and respiratory infectious diseases (23). The anti-inflammatory effect of diterpenoids isolated from the plant, as dehydroandrographolide, andrographolide, and neoandrographolide, were tested *in vitro* studies reporting the down-expression of genes involved in inflammatory cascade and the interfering COX and inflammatory cytokines (24). Moreover, the effect of andrographolide on the expression of inducibile NO synthase (iNOS) mRNA protein has been investigated, demonstrating that such molecule may reduce the expression of the protein both preventing the *de novo* synthesis and increasing the protein stability (25).

In addition, a clinical study was conducted using an herbal mixture of *A. paniculata*, comparing the extract with mesalazine, in patients with mild-to-moderately active ulcerative colitis. No significant difference in remission and clinical response between two groups has been reported, suggesting that the herbal extract *A. paniculata* may represent an effective alternative to mesalazine in the treatment of ulcerative colitis (26).

Our previous study showed that *Lentilactobacillus kefiri* SGL 13 exhibited anti-inflammatory and anti-cancer properties in LPS-treated HT-29 cells (27). Moreover, the anti-inflammatory properties of *L. kefiri* in the gut has been already demonstrated in mice (28, 29), proving that the administration of a strain of *L. kefiri* modulates the production of pro- and anti-inflammatory cytokines both downregulating the expression of pro-inflammatory mediators and increasing the anti-inflammatory molecules.

In the present study we aimed to exploit the combined action of *L. kefiri* SGL 13 and *A. paniculata*, in order to develop an effective alternative treatment for IBD without side effects. For this purpose, the safety of *Paniculin 13*™ and its efficacy in reducing the severity of IBD, was evaluated in C57Bl/6J mice fed a dextran sulfate sodium (DSS)-induced colitis diet.

## Materials and methods

### Bacterial strains and growth conditions

*Lentilactobacillus kefiri* SGL 13 (DSM 27331) was obtained from Sintal Dietetics Srl collection. The strain was grown in MRS broth (BD Difco, Franklin Lakes, NJ, USA) in aerobic condition and stored as stock cultures at −80°C in MRS broth supplemented with 15% (v/v) glycerol. In order to prepare *L. kefiri* SGL 13 biomass, lactobacilli were grown in MRS medium at 37°C and pH 5.5, in a 2 L fermentor (Omnitec srl, Milano, Italy) with an agitation speed of 200 rpm. Cells were harvested by centrifugation (17000 × *g*, 15 min at 4°C) and then lyophilized in a freeze-drier (Telstar Liobeta 3PS, Spain). After lyophilization, the number of viable cells/g was determined by plate count method.

### *Paniculin 13*™ formulation

*Paniculin 13*™ was composed of lyophilized *L. kefiri* SGL 13 (DSM 27331), dry extract of *A. paniculata* (*50*% andrographolide content) and inulin. Each daily dose per mouse consisted of SGL 13 (5 × 10^8^ CFU/kg bw) according to the formula for dose translation (30), *A. paniculata* (130 mg/kg bw) and inulin (1.25 mg/kg bw). Immediately before starting oral gavage procedure, the powder of *Paniculin 13*™ was suspended in a suitable volume of sterile phosphate buffered saline (PBS) (dose for each mouse = 0.2 ml/day). To assess the stability of *Paniculin 13*™, determination of the number of viable cells/vial of SGL 13, was carried out in triplicate by plate count method. The viability was determined on days 7, 14, 28, and 38; that is respectively after 0, 6, 20, and 30 days of *Paniculin 13*™ oral administration. The tolerance of SGL 13 to gastrointestinal conditions and its adhesion ability to epithelial cells HT-29, which are prerequisites for colonization and functional activity, were determined as described in a previous study (27).

Cells were harvested by centrifugation (17000 × *g*, 15 min at 4°C) and then lyophilized in a freeze-drier (Telstar Liobeta 3PS, Spain). After lyophilization, the number of viable cells/g was determined by plate count method.

### Animal experimental design

Animal experimental procedures were approved by Italian Ministry of Health (approval no. 229/2018-PR) and were performed in accordance with national regulation for care and use of laboratory animals and following internal procedures of Experimental Zooprophylactic Institute of Abruzzo and Molise (IZSAM) in order to minimize animal suffering and distress.

Forty 7 weeks-old C57BL/6J (WT) mice, all male, were obtained from Charles Rivers Laboratories (Wilmington, MA, USA). The number of animals per group was statistically determined and approved by the Italian Ministry of Health. On the basis of well documented safety profile of both *A. paniculata* (31–33) and *L. kefiri* (28, 34, 35) and to adhere to the 3Rs rules (Replacement, Reduction and Refinement), we reduced the number of animals required to assess the safety of *Paniculin 13*™ (G1 and G2). In order to demonstrate the efficacy of *Paniculin 13*™ in reducing the severity of IBD, we used a larger sample size for experimental groups treated with DSS (G3 and G4), improving the statistical power. Moreover, to maintain the initial assumptions about the minimal differences that the test would be able to highlight as significant, we included extra mice (one for each experimental group) to prevent unexpected deaths. Therefore, animals were randomized into 4 experimental groups: control group called G1 (*n* = 6 + 1), *Paniculin 13*™ group called G2 (*n* = 6 + 1), DSS group called G3 (*n* = 12 + 1) and DSS + *Paniculin 13*™ group called G4 (*n* = 12 + 1).

Mice were housed in collective enriched cages in a controlled environment (12:12 h light/dark cycle, 22 ± 2°C room temperature, 55 ± 5% humidity) and were allowed to acclimate for 7 days. Mice were transferred to individual cages only before fecal samples collection. The whole experimental procedure lasted 38 days. All animals were fed the same diet (2016 Teklad global diet, Envigo) with access to food and water *ad libitum*.

G1 and G3 received the vehicle PBS for 31 consecutive days (days 8–38), G2 and G4 received *Paniculin 13*™ for 31 consecutive days (days 8–38). *Paniculin 13*™ and PBS were administered by daily oral gavage (0.2 ml/day).

In addition, G3 and G4 received 1.5% (w/v) DSS (DSS for colitis, TdB Consultancy, Sweden, MW 40,000) in drinking water for 9 consecutive days (15–23) to induce colitis, followed by 15 days of washout (days 24–38). Fresh DSS solution was prepared every day and administered to mice. The average amount of DSS solution taken was recorded daily.

Tolerability and safety of *Paniculin 13*™ were assessed. The wellbeing and state of health of mice were observed during the entire experimental period by the animal technologists and care staff, using a clinical score sheet system. As shown in Table 1, a specific pain scale was used in order to minimize animal pain and distress. Different parameters such as general appearance, food/water intake, behavior, nest building, were assessed as indicators of health and welfare in mice (36–39). Body weight was measured weekly for mice of G1 and G2 throughout the whole experimental period and daily during the DSS treatment period for mice of G3 and G4. Moreover, during DSS administration clinical symptoms of colitis were monitored daily such as weight loss, diarrhea and presence of blood in the stool (40, 41).

**TABLE 1:** Scoring system for indicators of pain and distress in mice.

Appearance	Score
<5% weight loss	0
5–10% weight loss	1
11–15% weight loss	2
16–20% weight loss	3
>20% weight loss	HEP
Reduced grooming activity	1
Pinched skin/dehydration/slight piloerection	2
Strong piloerection	3
**Body functions**	**Score**
Dyspnea	2
Tachypnea	1
**Food and water intake**	**Score**
25–40% food/water intake reduction (for 3 days)	1
>40% food/water intake reduction (for 3 days)	2
>40% food/water intake reduction (for more than 3 days)	3
**Behavior**	**Score**
Reluctance to move, slightly hunched posture	1
Lethargy, apathy, hunched posture	2
Persistent immobility < 24 h	3
Immobility > 24 h	HEP
Vocalization on handling	1
Vocalization, tense and nervous on handling, aggressivity	2
Vocalization on moving/spontaneous	3
**Nest building**	**Score**
Nestlet slightly manipulated	1
Nestlet noticeably manipulated	2
No nest	3
**Altered intestinal function**	**Score**
Mild-soft stool and diarrhea	1
Presence of blood on the bedding	2
Rectal bleeding	HEP

**FIGURE 1:**
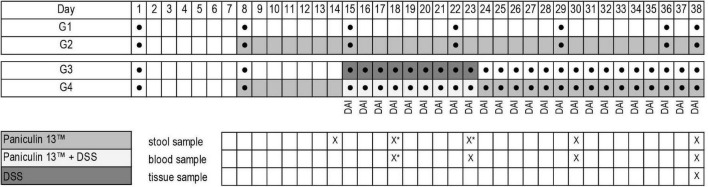
Experimental design of the study. Experimental groups (G1, G2, G3, G4); weight determination ●; *Paniculin 13*™ administration; DSS treatment; DAI score; collection date and time of stools, blood and tissues are indicated in the grid (x). *Stool samples on days 18 and 23 were collected only for mice of G3 and G4.

At the end of the study mice were euthanized by isoflurane inhalation followed by cervical dislocation, spleen and colon were collected and, respectively weighted and measured. Blood sampling (0.5–0.8 ml) were collected from each mouse. Blood glucose was measured by tail puncture using a glucometer (Accu-Chek^®^ Aviva, Roche Diagnostics, Germany) on days 23, 30, 38 for mice of G1 and G2 and on days 18, 23, 30, 38 for mice of G3 and G4. Total cholesterol (TC) triglycerides (TG) and high-density lipoprotein cholesterol (HDL-C) were measured at the end of the experiment (day 38) with enzymatic methods (42), using standard biochemical evaluations with Cobas c501 analyzer (Roche Diagnostic, Monza, Italy). The experimental design is depicted in Figure 1.

### Disease Activity Index (DAI)

The DAI was calculated by the sum of scores assigned for each DAI category (body weight loss, stool consistency and bleeding) based on scoring system of De Fazio et al. (41, 43). All parameters were scored from day 15 to day 38.

Stool samples were collected by placing a single mouse in an empty cage without bedding material for 15 to 30 min and fecal pellet were checked for color, consistency and presence of blood. The criteria for DAI scoring are described in detail below.

Body weight loss: body weight loss 0%, 0 points; 5–10%, 1 points; 11–15%, 2 points; 16–20%, 3 points; >20%, 4 points. Weight loss percentage was calculated, using the formula:


$$\left[\frac{\left(S\,t\,a\,r\,t\,i\,n\,g\,w\,e\,i\,g\,h\,t-C\,u\,r\,r\,e\,n\,t\,w\,e\,i\,g\,h\,t\right)}{S\,t\,a\,r\,t\,i\,n\,g\,w\,e\,i\,g\,h\,t}\right]\cdot 100$$

Stool consistency: formed, 0 points; mild-soft, 1 points; very soft, 2 points; watery stool, 3 points. Bleeding: normal color stool, 0 points; brown color, 1 points; reddish color, 2 points; bloody stool, 3 points. The DAI score ranged from 0 to 10 (total score).

### Histological evaluation of colon tissue

Mice (*n* = 7 for experimental group G1 and G2, and *n* = 8 for G3 and G4) were sacrificed on day 38 and the colons were excised immediately, rinsed in sterile PBS, fixed in 10% neutral buffered formalin, trimmed, and routinely processed for histology. After processing, samples were embedded in paraffin and 4 μm thick sections were obtained and stained with Mayer’s hematoxylin and eosin G (41, 43). Histological slides were then examined with a light microscopy.

### mRNA expression of MPO, TNFα, iNOS and COX-2 in colon tissues

Colon specimens were collected after sacrifice from each mouse and total RNA was extracted according to spin column (Qiagen, Milan, Italy). Tissue samples were homogenized by using TissueLyser II (QIAGEN, Milan, Italy) in ice cold commercial lysis buffer (RNeasy Micro Kit, Qiagen) supplemented with RNase-free DNase I (RNase-Free DNase Set, Qiagen) (44, 45). Reverse transcription-polymerase first-strand cDNA synthesis was performed by using maxima 1strand cDNA synthesis kit (Thermo, Life Technologies, Waltham, MA, USA). qPCR was then performed by using the Kit Thermo SYBR Green/ROX qPCR Master Mix (Thermo, Life Technologies, Waltham, MA, USA) and specific primers for selected genes. The analyses were performed with the 9,600 Biorad Real-Time PCR Detection Systems (Bio-Rad, CA, United States). The developed primer sequences have been listed in Table 2. PCR cycling conditions were as follows: 94°C for 5 min, 40 cycles at 94°C for 15 s, and 60°C for 30 s. Data were expressed as Ct values and used for the relative quantification of targets with the 2^–ΔΔCt^ calculation.

**TABLE 2:** Primer sequences used for real-time PCR.

Gene	Forward	Reverse	Accession number	Percentage identity %
TNFα	5′-CGTCGTAGCAAACCACCAAGT-3′	5′-TTGAAGAGAACCTGGGAGTAGACA-3′	X02611.1	100
MPO	5′-AGGAGGCCCGGAAGATTGTA-3′	5′-AGACATTGGCGATTCGAGGG-3′	NM_010824.2	100
IFNγ	5′-CCTGCGGCCTAGCTCTGA-3′	5′-CCATGAGGAAGAGCTGCAAAG-3′	NM_008337.4	100
iNOS	5′-GGCAGCCTGTGAGACCTTTG-3′	5′ -GCATTGGAAGTGAAGCTGTTC- 3′	M87039.1	100
COX-2	5′-GGGTTGCTGGGGGAAGAAATG-3′	5′ -GGTGGCTGTTTTGGTAGGCTG- 3′	M64291.1	100

TNF, tumor necrosis factor; MPO, myeloperoxidase; IL, interleukin; INF, interferon; iNOS, inducible nitric oxide synthase; COX, cyclooxygenases.

### Characterization of intestinal microbiota

Fresh stool samples were collected on days 14, 30 and 38 for G1 and G2 and on days 14, 18, 23, 30 and 38 for G3 and G4. Samples, were immediately stored at −80°C until microbiota analyses. Total bacterial DNA was extracted from 100 mg of pooled fecal material from each group, using QIAamp PowerFecal DNA Kit (QIAGEN, Manchester, UK) with modified protocol (46). Amplification of V3-V4 region of 16S rDNA was performed by PCR in 25 μL of final volume mix containing 12.5 ng of microbial DNA and 200 nmol/L of S-D-Bact-0341-b-S-17/S-D-Bact-0785-a-A-21 primers (47), carrying Illumina overhang adapter sequences. After two amplification steps and their respective purification steps as previously reported (42) amplicons were used to build libraries which were purified and successively pooled at equimolar concentrations (4 nM), denatured, and diluted to 5 p.m. Samples were sequenced on Illumina MiSeq platform using a 2 × 300 bp paired end protocol with 20% of PhiX as run control, according to the manufacturer’s instructions (Illumina, San Diego, CA, USA). Paired-end reads, obtained by sequencing, were analyzed using the 16S Metagenomics GAIA 2.0 tool (Sequentia Biotech, Barcelona, Spain, 2017; Benchmark of Gaia 2.0), which performs the quality control of the reads/pairs (i.e., trimming, clipping and adapter removal) through FastQC and BBDuk. The reads/pairs are mapped with BWA-MEM against the custom databases (based on NCBI).

### Statistical analyses

A non-parametric Mann–Whitney test was applied to verify any differences between 2 independent samples. Comparisons between k independent samples were made using the non-parametric Kruskal–Wallis test. If the Kruskal–Wallis test was significant, a *post hoc* analysis was performed to compare all possible pairs. Specifically, *post hoc* comparisons were carried out with the Dunn test (using the Bonferroni correction).

## Results

### Safety and stability assessment of *Paniculin 13*™

Prior to randomization, 3 mice were euthanized due to serious injuries. Thus, sample size of each experimental group was as follows: G1 (*n* = 7), G2 (*n* = 7), G3 (*n* = 13), G4 (*n* = 13).

**FIGURE 2:**
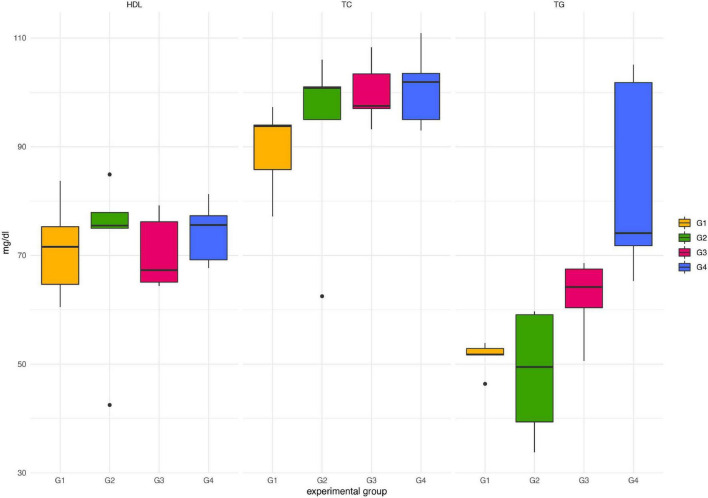
Lipid profile in mice of G1, G2, G3, and G4 at the end of the experimental period (day 38). TC, total cholesterol; HDL-C, high-density lipoprotein cholesterol; TG, triglycerides.

Throughout the experimental period, neither death nor adverse effects were observed in any of the animals treated with *Paniculin 13*™ (G2). *Paniculin 13*™ had no effect on blood glucose at the considered time points (data not shown). Regarding serum lipid profile (Figure 2), no significant differences in HDL-C and TC were found among groups. On the contrary, mice treated with only DSS (G3) and those treated with DSS + *Paniculin 13*™ (G4), showed higher TG values, compared to mice assigned to G1 and G2, but only the differences between G4 and G1 and G4 and G2 were statistically significant (*p* < 0.0083). To support the effectiveness of the association between SGL 13 and *A. paniculata* (*Paniculin 13*™), the viability and stability of SGL 13 in the presence of herbal extract was assessed *in vitro* by plate count. No significant differences in cell concentration were found among the considered time points, namely, day 1, day 14, day 28, and day 38 (10.07 ± 0.08; 10.12 ± 0.03; 10.10 ± 0.02; 10.04 ± 0.04). The results were expressed in log10/vial.

### Effects of *Paniculin 13*™ on clinical signs of colitis induced by DSS

**FIGURE 3:**
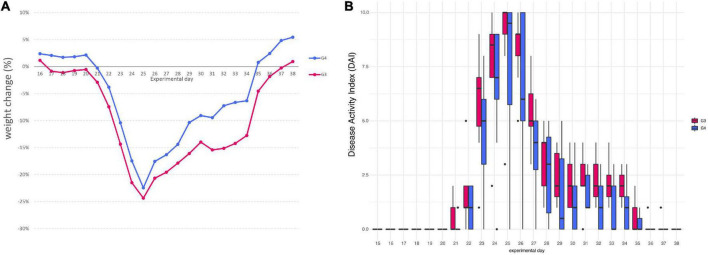
Variation in the average body weight gain percentage between mice of G3 and G4 **(A)**. Disease Activity Index (DAI) score of colitis in 1.5% DSS treated mice G3 and G4 **(B)**.

Mice assigned to DSS group (G3) began to lose weight on day 17, only after 2 days of DSS, with a maximum mean weight loss of 24.35% on day 25. On the contrary, in mice assigned to DSS + *Paniculin 13*™ group (G4), a first reduction in body weight was observed on day 21, after 6 days of DSS, with a peak of 22.45% on day 25. Mice of G4 showed less body weight loss, compared to those from G3, with a maximum weight loss difference on day 32. Mice of G3 continued to lose weight until day 37 and began to gain weight on day 38. On the other hand, mice of G4 showed a progressive increase in body weight from day 35 to the end of the experimental period, with a maximum mean weight gain of 5.45% on day 38. Overall, all the differences in weight changes between G3 and G4, were statistically significant (*p* < 0.05), throughout the whole experimental period (Figure 3A).

The weekly mean body weight of G1 and G2 was similar during the whole experimental period: G1 day 0 (21.87 ± 1.40), G1 day 15 (23.35 ± 1.07), G1 day 38 (25.33 ± 1.54) and G2 day 0 (20.93 ± 1.28), G2 day 15 (22.76 ± 2.32), G2 day 38 (25.89 ± 2.09).

Furthermore, all mice from G3 and G4 showed changes in stool consistency, 7 days after the beginning of 1.5% DSS assumption (day 22). Stool consistency scores were higher in mice of G3, compared those of G4, these last exhibiting only soft or very soft stools. A normal stool consistency was reached starting from day 30 for both G3 and G4. The presence of blood in feces was observed after 8 days of DSS (day 23) and was noticed until day 28 for both mice assigned to G3 and G4. Mice of G3 reached a maximum score of 3 for bleeding (bloody stools), while mice of G4 showed only reddish stools (score of 2). Overall, the most severe clinical signs were observed between day 23 and 26 for all mice, reaching a peak on day 25, with an average DAI score of 9 for mice of G3 and of 7.75 for mice of G4. DAI values were significantly higher in mice of G3 compared to corresponding values of G4 (*p* < 0.05), throughout the entire DSS treatment period (day 38). DAI score results are all displayed in Figure 3B.

Mice of G3 started to show mild signs of pain and distress (slight piloerection, reluctance to move, reduction in nest building activity) on day 19, only after 4 days of DSS. On the contrary, mice of G4 showed similar signs after 9 days of DSS. Symptoms of colitis became severe at the end of DSS treatment, peaking on day 25, when the maximum weight loss was observed. Consequently, 3 mice of both G3 and G4 died on day 25 and 2 mice died on day 28 in G3 and on day 31 in G4. The mortality rate was 41.7%. Nevertheless, mice that survived DSS treatment in G4, showed in general a more rapid improving of health conditions than those of G3. Overall, *Paniculin 13*™ seems to delay signs and symptoms of colitis, e.g., abdominal pain, stool consistency, presence of blood in stools, and weight loss.

### Effects of *Paniculin 13*™ on colon length and spleen weight

A non-parametric Mann–Whitney test was applied to verify any differences between 2 independent samples. The macroscopic changes associated with DSS-induced colitis classically include shortening of the colon (48), and splenomegaly (49). As colitis progresses and the epithelium begins to erode, the colon thins and becomes shorter and immune cells infiltrate the lamina propria (40). Changes in the inflammatory milieu driven by infiltration of immune cells results in an increased spleen weight. We found no significant differences in the mean colon length (cm) among control group G1, *Paniculin 13*™ group G2 and *Paniculin 13*™ + DSS group G4 (7.23 ± 0.46, 7.30 ± 0.50, 7.37 ± 0.58). On the contrary, the mean colon length of G3 was shorter (6.36 ± 0.63) than that observed in the other groups, even if this difference was not statistically significant. The mean spleen weight (mg) of mice in G1 and G2 was not significantly different, namely, 60.88 ± 9.65 and 76.83 ± 24.88, respectively. In comparison to G1 and G2, the mean spleen weight of DSS group was higher than that of DSS + *Paniculin 13*™ group (G3 = 125.01 ± 48.91; G4 = 100.51 ± 31.06), but only the differences between G3 and G1 were significant (*p* < 0.0083).

### Histological evaluation of colitis

Histopathological examination was carried out on small intestine and colon. Lymphoid aggregates in lamina propria were found in all mice as a part of gut associated lymphoid tissue (GALT). Lymphoid aggregates were classified as small when composed of less than 100 cells and large when composed of 100 or more cells.

**FIGURE 4:**
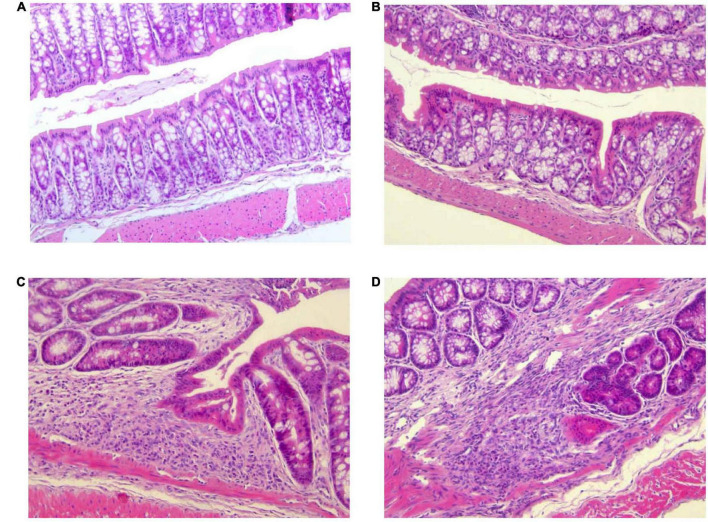
Differences in histological parameters during experimental colitis induced by DSS. Colon was collected at day 38 from mice of G1 **(A)**, G2 **(B)**, G3 **(C)**, and G4 **(D)**. Colonic sections were stained with hematoxylin and eosin and representative images were captured at 20× magnification. DSS treated mice are G3 and G4 groups.

Histopathological changes in the colon after DSS treatment are shown in Figure 4. Mice of control group (G1) and *Paniculin 13*™ group (G2) revealed a fully intact colonic epithelium with no inflammatory infiltration. The tissue damage induced by DSS, tended to be limited to the terminal colon. Mice of DSS group (G3) showed moderate cellular infiltration in the colonic mucosa (granulocytes, lymphocytes, macrophages) in association to flattening of the intestinal epithelium. On the contrary, mice of *Paniculin 13*™ + DSS group (G4) showed reduced post ulcerative areas with a lower inflammatory infiltration and a flattened intestinal epithelium. In both G3 and G4 signs of gut epithelial regeneration were seen at day 38, after 14 days of washout. Overall, supplementation with *Paniculin 13*™ seems to ameliorate colon tissue injury induced by DSS.

### Effects of *Paniculin 13*™ on mRNA expression levels of MPO, TNFα, iNOS and COX-2

**FIGURE 5:**
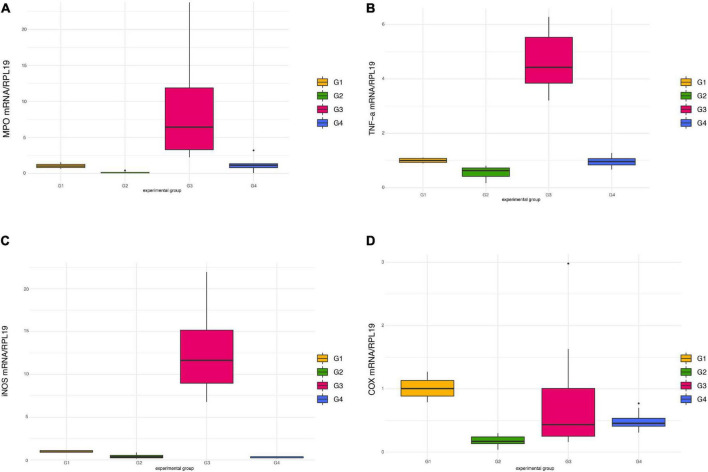
Pro-inflammatory mediators’ expression in colon tissue in mice of G1, G2, G3, and G4. mRNA levels of MPO **(A)**, TNFα **(B)**, iNOS **(C)**, and COX-2 **(D)** were all normalized against RPL-19 mRNA.

Exposure to DSS significantly raised the mRNA levels of MPO (Figure 5A) and TNFα (Figure 5B) in mice treated only with DSS (G3), an effect which was counteracted in G4 receiving DSS + *Paniculin 13*™, even if a significant difference between G3 and G4 was not reached. In both cases, only the differences between G3 and G2 were found statistically significant (*p* < 0.0083). A similar profile of expression was found in the case of iNOS (Figure 5C), where G3 showed significantly higher iNOS mRNA levels (*p* < 0.0083) compared to both G2 and G4.

Contrary to previous findings (41, 50), administration of DSS failed to induce COX-2 over-expression, in comparison to untreated mice (Figure 5D). Moreover, a decreased expression of this inflammatory mediator was observed in mice of G2 treated only with *Paniculin 13*™, namely, significant lower mRNA levels (*p* < 0.0083) compared to G1 (control).

### Intestinal microbiota modifications induced by *Paniculin 13*™

**FIGURE 6:**
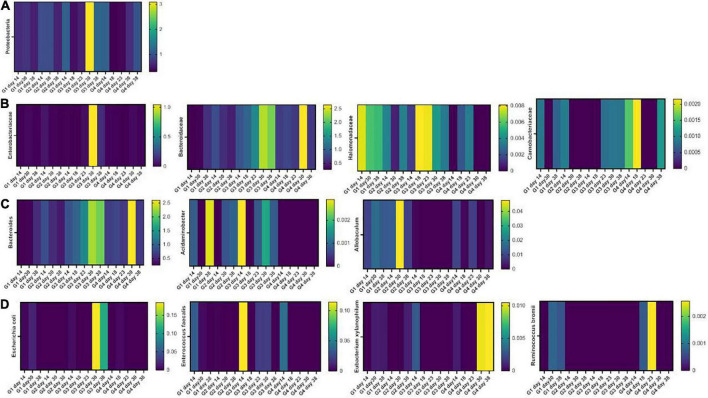
Heatmaps showing the fecal microbiota composition in mice of G1, G2, G3, and G4 at the phylum **(A)**, family **(B)**, genus **(C)**, and species **(D)** level.

*Firmicutes* and *Bacteroidetes* were the most abundant phyla in all samples. DSS induced an increment in inflammatory *Proteobacteria* which was found 1 week after DSS washout. This effect was mitigated by *Paniculin 13*™ supplementation (day 30, G3 = 3.09% and G4 = 0.68%) (Figure 6A). Going down the taxonomic scale, the families (Figure 6B) of *Enterobacteriaceae* and *Halomonadaceae*, both belonging to *Proteobacteria*, were found enriched in DSS-treated mice, but were reduced by *Paniculin 13*™ supplementation. In details, Enterobacteriaceae increased upon DSS treatment in G3 (day 23 = 0.03328%, day 30 = 1.04478% and day 38 = 0.13112%) while remaining less abundant in mice of G4 receiving DSS + *Paniculin 13*™ (day 23 = 0%, day 30 = 0.02845% and day 38 = 0.00698%).

As for *Halomonadaceae*, they were measured during DSS treatment in G3 (day 18 = 0.00812% and day 23 = 0.00792%) and in lower amounts in G4 (day 18 = 0.00216% and day 23 = 0.00341%), but after DSS washout, they only remained measurable in non-supplemented mice (day 30, G3 = 0.00434% and G4 = 0%; day 38, G3 = 0.00334% and G4 = 0%). In addition, *Bacteroidaceae*, previously found increased in mice treated with DSS (51), showed higher levels in G3 (day 18 = 1.03% and day 23 = 1.38%) as compared to G4 (day 18 = 0.77% and day 23 = 0.66%). Moreover, the *Carnobacteriaceae* family, which is expanded in IBD patients (52, 53), were detected in G3 during and after DSS exposure, but disappeared in G4 from day 23 to day 30. At the genus level (Figure 6C), a progressive increase in the LPS-producing *Bacteroides* was observed in DSS-treated mice (G3 day 18, 1.00% and G3 day 23 1.34%) in agreement with previous reports (54). This increase, however, was mitigated by *Paniculin 13*™ supplementation in G4 (day 18 = 0.73% and day 23 = 0.66%). Among other interesting changes, *Acidaminobacter*, which is increased in colorectal cancer patients (55), was detected in all time-point of G3 but was completely absent in *Paniculin 13*™ -supplemented G4. Further *Allobaculum*, a genus enhancing intestinal barrier by producing short chain fatty acids (56) was measured in G1 and G2, and it remained undetectable in G3 during DSS treatment and after washout, while being recovered in G4.

Finally, at the species level (Figure 6D), some pro-inflammatory and colitis-associated bacteria were only measured in G3 but not in G4 during and after DSS treatment, such as *Escherichia coli* (day 30, G3 = 0.18483% and G4 = 0%; day 38 G3 = 0.11359% and G4 = 0%), and *Enterococcus faecalis* (day 23, G3 = 0.01981% and G4 = 0%; day 30, G3 = 0.02083% and G4 = 0%). On the other hand, some beneficial species, producing short chain fatty acids, resulted enriched in the same comparisons, including *Ruminococcus bromii* which was absent in G3 but was measured in G4 during DSS treatment (day 18 and day 23) and *Eubacterium xylanophilum* which was depleted in both groups during DSS treatment but was restored only in DSS + *Paniculin 13*™ G4 during washout (day 30 and day 38).

## Discussion

In the last decade, there has been a growing interest in the use of probiotics for the treatment of intestinal disorders (57). Current IBD therapies, include treatment with COX-2 inhibitors, corticosteroids, immunomodulators, antibiotics, and biological agents. Long-term use of these conventional drugs can cause several severe side-effects, e.g., nausea, vomiting, headache, sickness, fever, rash, diarrhea, infections (58). Hence there is a need to discover effective and safe non-pharmacological remedies, as supportive approaches to IBD therapy, able to alleviate clinical symptoms and improve the quality of life of patients (13, 59). The beneficial properties of *L. kefiri* SGL 13 have been already investigated on human colon adenocarcinoma cells, HT-29. Upon treatment with SGL 13, the proteomic profile of HT-29 showed a total of 60 differentially expressed proteins compared to untreated cells, an effect apparently correlated with pro-apoptotic and anti-inflammatory pathways, highlighting a potential anti-tumor effect of SGL 13 (27). Evidence of efficacy is also available for the natural extract *A. paniculata*, in patients with IBD. The main known components of *A. paniculata*, the diterpene lactones, principally andrographolide and its derivatives, have been reported to exert anti-inflammatory properties through inhibition of the transcription factor NF-κB. NF-κB activation promoted the increased expression and synthesis of different pro-inflammatory mediators involved in the inflammatory response associated with IBD (60).

In the present investigation, we evaluated the impact of a combination of SGL 13 and *A. paniculata* herbal extract on a DSS-induced colitis mouse model, demonstrating that different IBD severity indicators were improved. In particular, the administration of *Paniculin 13*™ for 31 days reduced the weight loss percentage and the DAI score in DSS + *Paniculin 13*™ group (G4), that showed a reduced spleen weight and a longer colon tract compared to mice treated only with DSS (G3). Moreover, *Paniculin 13*™ ameliorated the general health status of G4, accelerating their recovery phase after DSS administration. The efficacy of *Paniculin 13*™ was supported by the histological examination of small intestine and colon sections, also since DSS + *Paniculin 13*™ group (G4) had reduced post ulcerative areas with a lower inflammatory infiltration and a flattened intestinal epithelium compared to DSS group (G3). These data positively correlate with mRNA expression in colon tissue of MPO, TNFα, iNOS, mediators which play a role in colitis induced by DSS (41). Tolerability and safety of *Paniculin 13*™ were demonstrated, since physiological parameters, such as blood glucose and lipid profile, and histological data were similar between control group (G1) and mice treated only with *Paniculin 13*™ (G2). Moreover, neither death nor adverse effects were observed in mice of G2 compared to G1. Concerning, the gut microbiota composition, in clinical trials, probiotics have been used as a supportive therapy for IBD, for the prevention of dysbiosis associated with long-term antibiotic or immunosuppressive therapies, as well as in the treatment of dysbiosis in patients with newly diagnosed IBD or with exacerbation of the disease (61, 62). Moreover, a number of different bacterial strains belonging to the genus of *Lactobacillus* have been used as probiotics for their ability to inhibit the growth of pathogenic bacteria (63). We found that *Paniculin 13*™ supplementation affected the microbial community, inducing favorable changes, such as the reduction of some inflammatory bacteria and the enrichment of some beneficial bacteria producing short chains fatty acids.

Although we are aware that the single administration with *A. paniculata* and *L. kefiri* SGL 13 alone is missing, which could have better clarified the synergistic effect due to the combined treatment, a possible limitation glimpsed, that reduced the statistical significance of our study, relies on the unexpected number of deaths in the groups receiving DSS (G3 and G4) associated with a more severe clinical phenotype, although we used the same protocol published by De Fazio et al. describing a mild model of colitis (41, 43). This investigation of course represents a preclinical assessment prior to developing further studies to confirm that the oral administration of *Paniculin 13*™ in patients with gut inflammation associated with dysbiosis is really effective.

Overall, this study highlighted the multifunctional properties of *Paniculin 13*™ supplementation, since dysbiosis, local and systemic inflammation, and tissue damage were simultaneously ameliorated. In conclusion, although further investigations are required, SGL 13 and *A. paniculata* could be considered a plausible supportive approach to IBD conventional therapy.

## Data availability statement

The sequencing datasets presented in this study can be found in the SRA (Sequence Read Archive) of NCBI, accession number: PRJNA899360 (https://www.ebi.ac.uk/ena/browser/home).

## Ethics statement

This animal study was reviewed and approved by Italian Ministry of Health (approval no. 229/2018-PR of 13/11/2017).

## Author contributions

LM, ERi, EB, and FF: conception and design of the study. LM, RS, and CP: data curation. LM, CM, and FF: formal analysis. LM, MR, MPV, and ERo: methodology. LM and EB: project administration. ERi: resources. VP, FP, and FF: supervision. LM, MR, RS, and FF: writing—original draft. ERi, EB, AC, CM, MPV, ERo, CP, and VP: writing—review and editing. All authors contributed to the article and approved the submitted version.
